# In the aftermath of teenage suicide: A qualitative study of the psychosocial consequences for the surviving family members

**DOI:** 10.1186/1471-244X-8-26

**Published:** 2008-04-21

**Authors:** Per Lindqvist, Lars Johansson, Urban Karlsson

**Affiliations:** 1 Division of Forensic Psychiatry, Department of Clinical Neuroscience, Karolinska Institute, Stockholm, Sweden; 2 Section of Forensic Medicine, Department of Community Medicine and Rehabilitation, Umeå University, Umeå, Sweden; 3 Department of Social Welfare, Umeå University, Umeå, Sweden

## Abstract

**Background:**

Studies of family reactions following teenage suicide are hampered by the psychological difficulties of approaching families and recruiting an unbiased sample of study subjects. By using a small but consecutive series of cases, we examined the qualitative aspects of loosing a teenage family member due to suicide. Such an understanding is important for future organisation of proper programs that provide professional support in the grief process.

**Methods:**

From a large project on teenage unnatural death in northern Sweden 1981–2000 (including 88 suicides), 13 cases from 1995 through 1998 were retrospectively identified and consecutively analysed. Ten families agreed to participate. The open interviews took place 15 to 25 months after the suicide. The information gathered was manually analysed according to a grounded theory model, resulting in allocation of data into one of three domains: post-suicidal reactions, impact on daily living, and families' need for support.

**Results:**

Teenager suicide is a devastating trauma for the surviving family and the lack of sustainable explanations for the suicide is a predominant issue in the grief process. The prolonged social and psychological isolation of the families in grief should be challenged. At the time of the interview, the families were still struggling with explaining why the suicide occurred, especially since most suicides had occurred without overt premonitory signs. The bereaved family members were still profoundly affected by the loss, but all had returned to an ostensibly normal life. Post-suicide support was often badly timed and insufficient, especially for younger siblings.

**Conclusion:**

Family doctors can organise a long-term, individually formulated support scheme for the bereaved, including laymen who can play a most significant role in the grief process. There is also a need for better understanding of the families who have lost a teenager whom committed suicide and for the development and testing of treatment schemes for the bereaved family.

## Background

Committing suicide in the teenage years can be perceived as the ultimate rejection of family, of significant others, and of society. The family involved will search for premonitory signs and clues to make sense of the tragedy [1]. Self-reproach for inadequate parenting and neglect in foreseeing and preventing the tragedy is the rule [2], and the tragedy is further complicated by the social stigma in society [3].

The bereavement process after a suicide may differ qualitatively from other types of losses [4-6] with prolonged reactions of grief and loneliness [7], greater feelings of shame [2], and perhaps most importantly, the prolonged search for the motive behind the suicide. It has been suggested, however, that suicidal deaths have more similarities than differences with accidental deaths. Both occur suddenly and the survivors are given no chance to say farewell, they learn of their loss by surprise, and they are not given any chance to adjust to a life without the deceased [8].

Studies of family reactions following teenage suicide are hampered by the psychological difficulties of approaching the families and recruiting an unbiased sample of survivors. Most interview studies involve samples that are either compromised by a high attrition rate, or based on survivors who organise and actively work through the crisis in support groups for suicide survivors [8].

This study derives from a larger research project on teenage unnatural deaths [9] in which all teenage suicides in northern Sweden from 1981 through 2000 (n = 88) were identified and the results were based on file material alone. In the next step, we used file material, but we also interviewed professionals involved in the aftermath of teenage suicides [10]. In the third step, we used the same mixed method to investigate suicide survivors, and this time we interviewed parents and siblings. An inductive method based on grounded theory [11,12] was chosen for the qualitative part since deductive methods, such as a case-control study, would not properly appreciate cultural and situational circumstances and, subsequently, not catch the cultural forces and motives of an event of this kind [13]. The quantitative part of the results was built on file material generated at the Department of Forensic Medicine, Umeå, Sweden.

This study's main purpose was to interview surviving family members that had lost a teenager by suicide to increase the understanding of the circumstances that these families are living under and to generate hypotheses to be tested in future research. The way of living in the northern rural areas of Sweden differs from the southern parts of Sweden and from other more densely populated areas studied previously.

## Methods

### Subjects

Teenage suicides (n = 13, age 13 through 19) occurring in the four northernmost counties of Sweden in 1995 through 1998 were retrospectively identified through the files of the Department of Forensic Medicine, Umeå. No additional cases were found after consulting the national Cause-of-Death Register [10]. Since all suicide victims financially depended on their families and none of the deceased had established a family of their own, our definition of a family was the persons living in the same household as the deceased at the time of the suicide. Persons included by this definition were biological parents, stepfathers, stepmothers, and siblings.

For six cases, both biological parents were interviewed. Two interviews involved the biological mother and a stepfather, and two interviews were conducted with either the biological mother or father. Siblings were present in five of seven possible cases (three couples had lost their only child). Three families chose not to participate; one family "wanted to forget", another was upset about the proposal, and one did not respond at all. Thus the surviving families of ten suicide victims were included.

The demographic characteristics of the suicides and the nature of the suicides were similar to 15 other cases from the study area (1993–1995) [10]. Two subjects in the present study were in psychiatric out patient treatment and two were seeing a school counsellor at the time of the suicide. The remaining six victims were as far as we have established from the interviews and the file material unknown to social and health care services.

The predominantly rural study area comprised 902,000 inhabitants in 1998 [14]. The overall national incidence of suicide 1998 was 21/100,000 [14]. The mean annual suicide incidence between 1993 and 1995 among teenagers in the study area was 8/100,000 [10], an incidence figure that is similar to national figures [14].

### Procedure

The overall strategy was to gather qualitative information using a grounded theory-like model with unstructured interviews and quantitative data from official documents (autopsy report, police report, and medical records, if present) at the Department of Forensic Medicine, Umeå. The rationale behind choosing this design was to increase understanding rather than revealing causal relationships or testing any pre-defined hypothesis. The grounded theory was originally described by Glaser and Strauss in 1967 [11]. The authors subsequently developed the grounded theory in different directions [15,16]; however, in this paper, we are relying more on Glaser's version [16].

In advance, the interviewer (PL, specialist in Child and Adolescent Psychiatry and Senior Consultant in Forensic Psychiatry) had studied the file material to determine the age and sex of the victim and which suicide method had been used. This was made in respect of the families as we thought they would expect the interviewer to have some basic knowledge of the deceased.

The method used requires mutual trust and identification with the respondents and critical self-reflection. As opposed to structured interviews, the researcher is "allowed" to answer questions if they help the interview and the respondent. Notes were taken throughout the interview, but we did not use any audio or video recording devices, which is in line with the grounded theory method [11,12]. If we had recorded the interviews, we would have had the possibility to check important notes taken during the interview against the tapes. We did decided, however, after intense discussion and reflection, not to use audio/video recording since we believe that such arrangements would have hampered the interviews. The sensitivity of the interview themes evoked emotions that would have been difficult to express in front of an audio/video recorder. The same method was used when we interviewed professionals involved in teenager suicides [10] where we managed to come very close to the investigating police officers who openly expressed their emotional concern, even with tears.

In the first six cases, we telephoned a professional who had been involved in the suicide investigation according to the police records to examine the feasibility of conducting a research interview with the family. This step of caution turned out to be unnecessary and was later dispensed with. Instead, we sent a letter with a brief description of the study directly to the family asking for a research interview. The letter was addressed to the parent mentioned in the police report or to both if that was the case. The letter also informed the recipients that they would be phoned in about two weeks providing an opportunity to obtain more information. If the family agreed to participate, a date and venue for the interview was arranged. It was left to the parents to decide which family members should participate.

The mean interval between the suicide and the first interview in this retrospective study was 17 months (range 15–25 months). All families wanted to be interviewed in their homes. The mean duration of the home visits was 2 1/2 hours (range 1 1/2–6 hours).

Our ambition was to come as close to the families as ethics and professional concern allowed. The conversations were permitted to find their own way. Because this is a hypothesis generating study, all interviews were started and ended with a broad research question: "Tell me about NN (the deceased)" and were closed with the questions: "Do you want to tell me anything else that we have not talked about?" and "What do you think of this interview?"

Without being asked, all respondents showed the interviewer the victim's bedroom with its various possessions and several families also offered to show the interviewer the site of the suicide if the suicide took place in the home.

Hard data and subjective reflections were transcribed immediately after the interview and, in most cases, for some time afterwards, described in grounded theory as "memoing". Each interview was reviewed in a session with a senior psychologist where the interviews were re-analysed and subsequently transcribed.

### Data analysis

The interviews were distressing even for the interviewer and there was a need for integration and psychological distance. Later, the interviews were analysed by repetitive perusal and consideration, resulting in the allocation of data into one of three domains: the post-suicidal reactions including the issue "why?"; the impact of the suicide on daily living; the families' need of support after the suicide. The recurrent and/or prominent findings within each domain were accounted for separately. The qualitative part of the study concerning the data collection, note-taking, coding, and memoing was very close to the grounded theory model as described by Glaser [16], which is also described in a more comprehensive form by Dick [12].

The procedure was approved of by the Research Ethics Committee of Umeå University, given that no single case could be identified at publication. Therefore, this account is deliberately vague about details of individual cases.

## Results

### The search for the "why?"

The most poignant theme of the interviews was the search for the "why?" which still preoccupied most of the parents. This search was even more salient in the eight cases where the suicide had come unexpectedly, "like a bolt from the blue." Most of these teenagers had disguised their suicidal ideation not only from their families but also from other adults and peers.

Almost all parents expressed anger at being deceived, a deception that denied them the opportunity to provide parental support. Simultaneously, they aired remorse for being angry with someone who obviously had been so lonesome and desperate. The "forbidden" anger appeared to be quite a problem.

Coming to terms with the "why?" was seemingly easier in the two cases where the risk of suicide had been evident beforehand. Some relief was even expressed by these families since they had long been living in suspense. This sense of relief was not easily admitted.

Most teenagers and their families had lived a pro-social life, which increased the confusion. Several of the deceased had, for example, been informal leaders for their peers and were average or high achievers in school, "a pride to any parent". Nonetheless, they had faced problems such as a broken love affair, fear of pregnancy, or difficulties with friends. Just before the suicide, however, the emotional turmoil of the teenagers had in most cases seemingly petered out, which, ironically, had put their parents at ease.

The families found it difficult to understand why "common teenage problems" had transformed into a matter of life or death. In hindsight, they reproached themselves for this ignorance, but acknowledged the dilemma of being a parent to a teenager, finding the optimal balance between active intervention and respect for their child's own way. A common remark was "How could we have known?"

Six teenagers had written suicide notes, but these neither enlightened nor soothed the parents. Common messages - such as "I love you," "Forgive me," "I cannot go on living," "Don't be angry with me," "Try to forget me" - shed little light on the issue. Two letters yielded instructions to pass on personal belongings to siblings, an uncomfortable inheritance for many of these.

### Impact on daily life

Although a long time had passed since the suicide, the families were still struggling to move on. Those who had lost their only child appeared to have the greatest difficulty. All had returned to the routine activities of daily life with much anguish and slowly receding anxiety. Almost all parents thought it impossible to ever return to "normality" and the ambition was reduced to manage another day. None had experienced one full day without thinking of the deceased. Some parents had entertained the idea of committing suicide themselves, but decided against this course of action being all too familiar with the consequences for others.

All families were relatively indifferent to "what other people would think." Their own feelings of guilt, shame, and self-reproach overwhelmed fears of social stigmatisation. Several families admitted previous prejudice towards families whose children had embarrassed their families. This was, paradoxically, a helpful experience.

### Post-suicidal support

All but one parent told of the immediate shock, disbelief, confusion, and outrage that lasted for months. Those who found early support from their extended family, friends, or close members of their church were grateful to these people. Those who found themselves subjected to the attention of self-invited professional helpers were more critical.

In the post-shock phase, a minority of the respondents had been or was subjected to professional counselling, and others were helped by clergy. Laymen from the Swedish organisation for suicide bereaved (SPES) played a significant therapeutic role in some cases; however, many families found themselves alone with the grief too soon after the suicide. They articulated a need to work the crisis over when the level of anxiety was endurable. Generally speaking, the families' relationships with both friends and community members were, at the time of the interviews, dissatisfying. They felt that others expected them to forget and get on with life.

In the cases where younger siblings were present at the interview, the parents were most pleased with their silent participation. Few adults, professionals, or others had previously succeeded with involving these children in sharing thoughts about the loss. According to data obtained from some random follow-up contacts, the research interviews had been most beneficial for these siblings.

## Discussion

### Psychological consequences

The most poignant issue coming out of this study was the parents' struggles to come to terms with the "why?" to find a rational answer to why their child chose to commit suicide, an answer that would make it possible for the bereaved to come to rest and go on with their own lives. The families appeared to increasingly turn to the question of "what for?" rather than "why?" suggesting that the suicide could not be explained but perhaps be seen as meaningful in some way. If there are no answers, why ask questions? Parents with other children to care for had little choice but to go on, at least physically if not spiritually.

The "search for the meaning" often plays a vital role in the struggle of bereaved persons to adapt to losses [17]. However, there seems to be a difference between unexpected losses and losses due to a known disease in which the bereaved have had a chance to adjust to the forthcoming death of a loved one. In the first case, there is some evidence that grief therapy is efficient and safe; in the other case, it can even be deleterious [17]. Neimeyer suggests that when grief therapy is offered, it must attend to the profound challenges to clients' (inter-) personal systems of meanings brought about by tragic loss and facilitate the survivors' own struggle to find significance both in the death and their ongoing lives [17].

Most teenagers who commit suicide do not express suicide feelings or otherwise hint about the forthcoming suicide. This silence was also recognized when we interviewed professionals involved in teenager suicides [10]. Although most of the suicides occurred in rural and depopulated areas where the social control often is pronounced, information about the families concerned was often sparse [10].

Some teenagers had written a suicide note, but these notes were of little help in understanding the suicide. One can only speculate whether the stereotyped suicide messages reflect the crux of the matter; viz., a teenager who can effectively relate his/her predicament to others may be less inclined to consider suicide.

Another salient feature of the psychological state of the families was the sense of helplessness. As expressed by Dunne [18], families are "propelled into uncharted waters without benefit of either a rudder or a pilot." Since the families had no previous experiences of the kind and no conceptual framework to relate to and subsequently no words to cover the event, the families were altogether disempowered.

### Impact on daily life

Although all families had returned to everyday activities, they were still preoccupied by the loss. To avoid "bothering" others, they had withdrawn from casual socialising. This can be understood from several perspectives. The sense of being deceived results in low self-esteem and nourishes feelings of inferiority and shame. Aggressive feelings towards the teenager and his/her unilateral decision to end their relationship are likely to encourage feelings of guilt. Prejudiced peers aggravate the situation, and, as described by Rudestam and Imbroll [19], the family "will subsequently face the added psychological stress of societal blame and reduced social support. To the extent that children's suicidal behaviour reflects motives of punishment and revenge directed at unresponsive parents, their actions may, from one perspective, be deemed successful." Metaphorically, many of the bereaved appeared to be imbedded in silence.

Some parents had thought of committing suicide themselves, but decided not to since they were all too familiar with the consequences for the bereaved. Nevertheless, caution is justified in the aftermath of suicide [20]. The rate of suicide in families of suicide victims is twice as high as in families of comparison subjects, and a family history of suicide is a significant risk factor independent of severe mental disorder [20]. This knowledge can be used for interventional purposes, but one must acknowledge that men and women often respond differently to typical bereavement interventions; that is, gender differences may need to be taken into account when designing interventions [21], a finding supported by Murphy et al. [22].

### Post-suicidal support

In a setting similar to ours, Dyregrov (2002) concluded that parents clearly indicate that support from their social network is crucial but insufficient, and the parents' want and need more assistance from local authorities than is provided. The bereaved asked for outreach and immediate assistance from trained personnel, long-term follow up, information and care for surviving children [23].

Our results confirm that the post-suicidal support was sometimes ill timed and randomly provided. The needs during the first phase of shock, denial, and overt confusion are comfort, familiarity, and provision of basic needs such as food, paying bills, etc. These needs, however, were sometimes unmet and replaced by psychotherapeutic penetration or by anxiety of bystanders visiting the families. Later, as the initial upheaval abated, the need to talk was often adequately provided by mental health professionals, clergy, or fellow suicide bereaved from the SPES organisation. However, as reported in other studies, the long-term needs of reflection appeared to have been underestimated, especially by health professionals [5,24]. Clergy, familiar with spiritual agony, and members of other suicide stricken families were less afraid of standing close by for an extended time.

Unfortunately, younger siblings had received little help to work the crisis through. These children are more likely to be burdened than older siblings [25,26] and need more time, more persistence, and an uncompromising readiness by the adult to deal with the most difficult questions. They will resist any volunteer, not giving the adult world a second chance if once they decide to seal their lips. Parents who have lost a child to suicide are often devastated for a long time having a reduced capacity to care for siblings who, therefore, need attention and support from resources outside the family [26]. The help needs to be directed as direct help to siblings and parents, as well as to the family as a whole [26]. The importance of including parents in the treatment of childhood traumatic grief is also pointed out [27]. Group therapy for bereaved siblings appears to be an appropriate method of helping [28,29]. During the interviews, the siblings were not very forthcoming but appeared very attentive. Because they did not verbalise their discomfort, their grief process cannot be accounted for.

It is not self-evident, however, that post-suicide support is a medical issue, although this is what survivors expect according to a report from the USA [30]. The medical paradigm may be unfit for issues entering the realms of spirituality and existential difficulties. Providing comfort, warmth, sympathy, and forgiveness is linked to humanity and not to training. Consequently, the main task for a responsible person from the health services (e.g., a family doctor) is to provide support for a bereaved family and to organise and create space for others such as laymen, members of the SPES (suicide survivors organisation) organisation, clergy, nurses, counsellors, etc. Such leadership requires the skill to know the changing needs of a mourning family. Most importantly, in instances like this, the survivors need people who know that "being" is more essential than "doing". "A bereaved parents' recommendation" has been issued [24] that is based on experiences from parents that have been interviewed.

### Validity

We believe that this explorative study meets a request to employ new research perspectives on teenage suicide [31]. The study design is inductive and relies heavily on the authenticity and quality of the interview situation, which is impossible to replicate. Due to the flexible method of data gathering and data analyses, we have tried to be cautious in terms of interpretation, yet we believe that this kind of descriptive and "naïve" anthropological approach can yield valuable insights.

A more methodologically structured approach would have secured the internal reliability but restricted the space for the parents to tell their story [24]. The families were at the time of the interviews more or less embedded in silence and thus eager to tell of their life situation when the purpose of the study and the integrity of the interviewer was accepted. The openness of the interview told the families that their views, whatever they were, were more important than testing a predetermined research hypothesis. This possibly elicited information that otherwise may have been veiled.

A follow-up study of bereaved parents' experience of research participation has shown that 100% of the subjects experienced the participation as positive or very positive and the parents linked their positive experiences to the fact that they were allowed to express their story [24]. They also expressed a hope that their story might help others [24].

The external validity can also be questioned. The study area carries specific features, with its mostly rural population that lags behind the life in the fast lane of urban cities. It is likely that some types of teenage suicide are underrepresented in this small series. A similar study of an urban area where norms, family structure, and drug use are different would possibly yield other results. Nonetheless, the question of living or dying is as old as mankind and similarities rather than differences are more likely.

### Further research

A cross-sectional study like this cannot claim to capture a mourning process, which by definition is characterised by change. The impact of reality and the sequence of various psychological defence mechanisms vary over time. With few exceptions [25,32], survivors of teenage suicide have not been subject to long-term and re-iterated longitudinal analyses. Without such studies, in different cultural contexts, it is not possible to tailor an optimal support scheme for families who have lost a teenager by suicide. Such an analysis should incorporate younger siblings who evidently are difficult to help.

It makes sense to study teenage suicide separately, apart from suicide committed by young adults. Mixing young adults, where suicide is more common, with teenagers, where suicide is infrequent, can confuse the overall picture. These two groups differ significantly in terms of psychological and social development and should thus be handled as related but separate phenomena.

## Conclusion

A teenager suicide is a devastating trauma for the surviving family as a whole and the absence of sustainable explanations to the suicide is a predominant issue in the grief process. The prolonged social and psychological isolation of the families in grief should be challenged and, e.g., a family doctor with a keen ear should organise a long-term, individually formulated support scheme for the bereaved. Laymen and clergy can also play a most significant role in the grief process.

There is a need for better understanding and treatment schemes for families who have lost a teenage family member in suicide, and especially for the younger siblings who often are forgotten.

## Competing interests

The author(s) declare that they have no competing interests.

## Authors' contributions

PL was responsible for the planning of the study, for the interviews, and for writing the first drafts of this paper. LJ took part in setting up the study and identified the cohort and the sample. He also collected collateral data, interviewed investigating police officers, and revised the drafts. UK has provided the team with expertise on qualitative research and revised the manuscript.

## Pre-publication history

The pre-publication history for this paper can be accessed here:


